# Door-in to door-out times in acute ST-segment elevation myocardial infarction in emergency departments of non-interventional hospitals: A cohort study

**DOI:** 10.1097/MD.0000000000020434

**Published:** 2020-06-05

**Authors:** Sandrine Clot, Thomas Rocher, Claire Morvan, Mathieu Cardine, Mohamed Lotfi, Julien Turk, Pascal Usseglio, Vincent Descotes-Genon, Gerald Vanzetto, Dominique Savary, Guillaume Debaty, Loic Belle

**Affiliations:** a Emergency Department and Emergency Medical Service, Metropole Savoie Hospital, Chambery; b Emergency Department and Emergency Medical Service, Annecy Hospital, Annecy; c Biostatistician, RENAU (Reseau Nord Alpin des Urgences), Annecy; d Emergency Department and Emergency Medical Service, Grenoble University Hospital, Grenoble; e Department of Cardiology, Annecy Hospital, Annecy; f Department of Cardiology, Metropole Savoie Hospital, Chambery; g Department of Cardiology, Grenoble University Hospital, Grenoble, France.

**Keywords:** door in door out, emergency department, percutaneous coronary intervention, ST-elevation myocardial infarction, thrombolysis

## Abstract

In France, one in eight patients with acute ST-segment elevation myocardial infarction (STEMI) is admitted direct to an emergency department (ED) in a hospital without percutaneous coronary intervention (PCI) facilities. Guidelines recommend transfer to a PCI center, with a door-in to door-out (DI–DO) time of ≤30 min. We report DI–DO times and identify the main factors affecting them.

RESURCOR is a French Northern Alps registry of patients with STEMI of <12 h duration. We focused on patients admitted direct, without prehospital medical care, to EDs in 19 non-PCI centers from 2012 to 2014. We divided DI–DO time into diagnostic time (ED admission to call for transfer) and logistical time (call for transfer to ED discharge).

Among 2007 patients, 240 were admitted direct to EDs in non-PCI centers; 57.9% were treated with primary angioplasty and 32.9% received thrombolysis. Median (interquartile range) DI–DO time was 92.5 (67–143) min, with a diagnostic time of 41 (23–74) min and a logistical time of 47.5 (32–69) min. Five patients (2.1%) had a DI–DO time ≤30 min. Five variables were independently associated with a shorter DI–DO time: local transfer (mobile intensive care unit [MICU] team available at referring ED) (*P* = .017) or transfer by air ambulance (*P* = .004); shorter distance from referring ED to PCI center (*P* < .001); shorter time from symptom onset to ED admission (*P* = .002); thrombolysis (*P* = .006); and extended myocardial infarction (*P* = .007).

In view of longer-than-recommended DI–DO times, efforts are required to promote urgent local transfer and use of thrombolysis.

## Abstract 2

Supplemental Digital Content is available in the text

## 1 Introduction

Acute ST-segment elevation myocardial infarction (STEMI) is a major cardiovascular emergency.^[1]^ STEMIs affect 120,000 patients in France each year; acute mortality is high, and early reperfusion improves prognosis.^[1–4]^ For example, in-hospital and 30-day mortality rates of 4% to 6% and 6% to 8%, respectively, have been reported in a recent French study of patients with STEMI who were treated in mobile intensive care units (MICUs) during 2009 to 2013.^[5]^ Early reperfusion (door-to-balloon time ≤90 min) has been shown to significantly improve both short- and long-term outcomes in meta-analyses.^[6,7]^

In France, contacting the emergency call center gives dedicated access to the prehospital Emergency Medical Service (EMS). When STEMI is diagnosed by the EMS, patients receive prehospital care in MICUs en route to centers with percutaneous coronary intervention (PCI) facilities. This immediate activation of reperfusion pathways reduces treatment delays and patient mortality.^[8–10]^ However, one-third of STEMI patients are admitted direct to an emergency department (ED) (e.g., by personal transport or regular ambulance, so they do not benefit from prehospital MICU treatment), and some of these hospitals lack PCI facilities.^[2,11]^ The European Society of Cardiology (ESC) recommends urgent transfer to a PCI center, with a door-in to door-out (DI–DO) time ≤30 min (from arrival at, to discharge from, the referring ED).^[1]^ Thrombolysis must be used if reperfusion is delayed by >120 min.^[1]^

Although US and Canadian studies have reported DI–DO times,^[12–19]^ in France, where prehospital organization is different, DI–DO times have not yet been published. We sought to report DI–DO times in a French registry and identify the main factors affecting them.

## 2 Methods

We analyzed data from patients with STEMI of duration <12 h (acute STEMI) enrolled in RESURCOR—an ongoing registry in the French Northern Alps started in 2002,^[20]^ with a reported accuracy of 84%.^[21]^ The registry listed initial characteristics (date/hour of admission to ED, medical history, type of/delay to reperfusion) for all STEMI patients; the data were recorded by the treating physician.^[22]^ All patients provided oral consent. Patients were enrolled prospectively; additional data were recorded retrospectively from the STEMI QUAL project, which was approved by the French administrative regulatory body (Commission Nationale de l’Informatique et des Libertés, Paris, France, No DR-2015-642). The STEMI QUAL project was supported by a grant from the French Ministry of Health (PREPS 2014 14–0040). The registry area encompasses three main French administrative departments in the French Northern Alps, with 19 EDs without PCI facilities and five PCI centers (see map in Supplemental Digital Content 1)”.

We analyzed data from patients with acute STEMI who were admitted direct to 19 EDs of non-PCI centers (January 2012 to December 2014) and were subsequently transferred to a PCI center. These patients arrived at the ED by personal transport, regular ambulance, or with the fire service. We excluded patients who received prehospital medical care in a MICU, patients hospitalized for another condition and who subsequently developed STEMI, patients admitted direct to an intensive care unit, and patients who had an early cardiac arrest before ED admission. For this analysis, the MICU team (medical team that does the transfer) was distinguished from the ambulance (means of transport); the possibility of transfer from a non-PCI center to a PCI center depended on the availability of a MICU team and ambulance at each ED (see Supplemental Digital Content 2, for details of the included EDs). Six air ambulances were available.

DI–DO time was defined as the time from arrival at, to discharge from, the initial ED in the non-PCI center (the “referring ED”). DI–DO time was divided into “diagnostic time” (from admission to the ED to transfer decision) and “logistical time” (from transfer decision to discharge from the ED). The transfer decision time was taken as the time of the physician's call to the emergency call center. The transfer was then organized by the emergency call center. The transfer was “local” if the MICU team was available at the referring ED and “non-local” if they were from another center.

### 2.1 Statistical analysis

Continuous variables are expressed as median (interquartile range [IQR]) and categorical variables as count (percentage). Student's *t* test or the Wilcoxon test were used to compare continuous variables; the chi-square test was used to compare categorical variables. Identification of DI–DO time explanatory variables was done using multivariable analysis (linear regression) (see additional statistical methods in Supplemental Digital Content 3). All tests were two-tailed; *P* < .05 was considered statistically significant. Statistical analyses were performed using R software, version 3.2.2 (R Core Team, 2017).

## 3 Results

Among 2007 STEMI patients enrolled in the RESURCOR registry during 2012–2014, 425 (21.2%) were admitted direct to an ED, of which 240 (56.5%) were admitted to an ED without PCI facilities (see Supplemental Digital Content 4, for the study flow chart). Most of the remainder received prehospital care by a MICU team, that is, followed the EMS pathway (70.6%). The characteristics of patients admitted direct to an ED in a non-PCI center were generally similar to those admitted to an ED in a PCI center (Table 1). Patients admitted direct to an ED had longer delays than those who used the EMS (see Supplemental Digital Content 5, for characteristics by initial care).

**Table 1:**
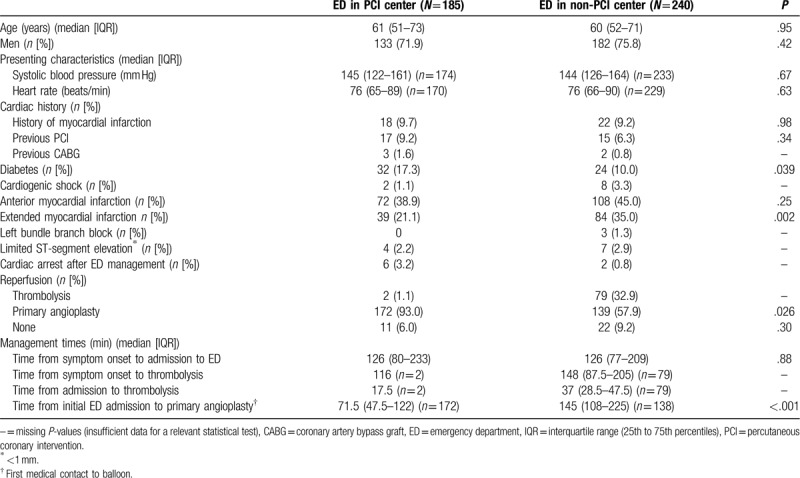
Baseline and procedural characteristics according to availability of percutaneous coronary intervention facilities.

### 3.1 Population

In our population of 240 patients admitted to the ED in a non-PCI center, 75.8% were men and the median age was 60 years (Table 1). One-third of patients (32.9%) received thrombolysis in the ED of a non-PCI center; in 51 of these patients (64.6%), thrombolysis took place within 30 min. Only 43.0% of our thrombolysed STEMI patients had signs of reperfusion on arrival at the PCI center. Primary angioplasty was undertaken in 139 patients (57.9%); among them, 45 patients (32.4%) had it performed within 120 min (median [IQR] first medical contact to balloon time of 145 min [108–225]) (Table 1). Ten patients (4.2%) died during hospitalization.

Overall, 109 patients underwent a local transfer to a PCI center (45.4%), 97 underwent a non-local transfer to a PCI center (40.4%), and 34 were transferred by air ambulance (14.2%) (Supplemental Digital Content 2).

### 3.2 Outcomes

Only five patients (2.1%) had a DI–DO time of ≤30 min. Median (IQR) DI–DO time was 92.5 (67–143) min, divided into a diagnostic time of 41 (23–74) min and a logistical time of 47.5 (32–69) min.

**Figure 1:**
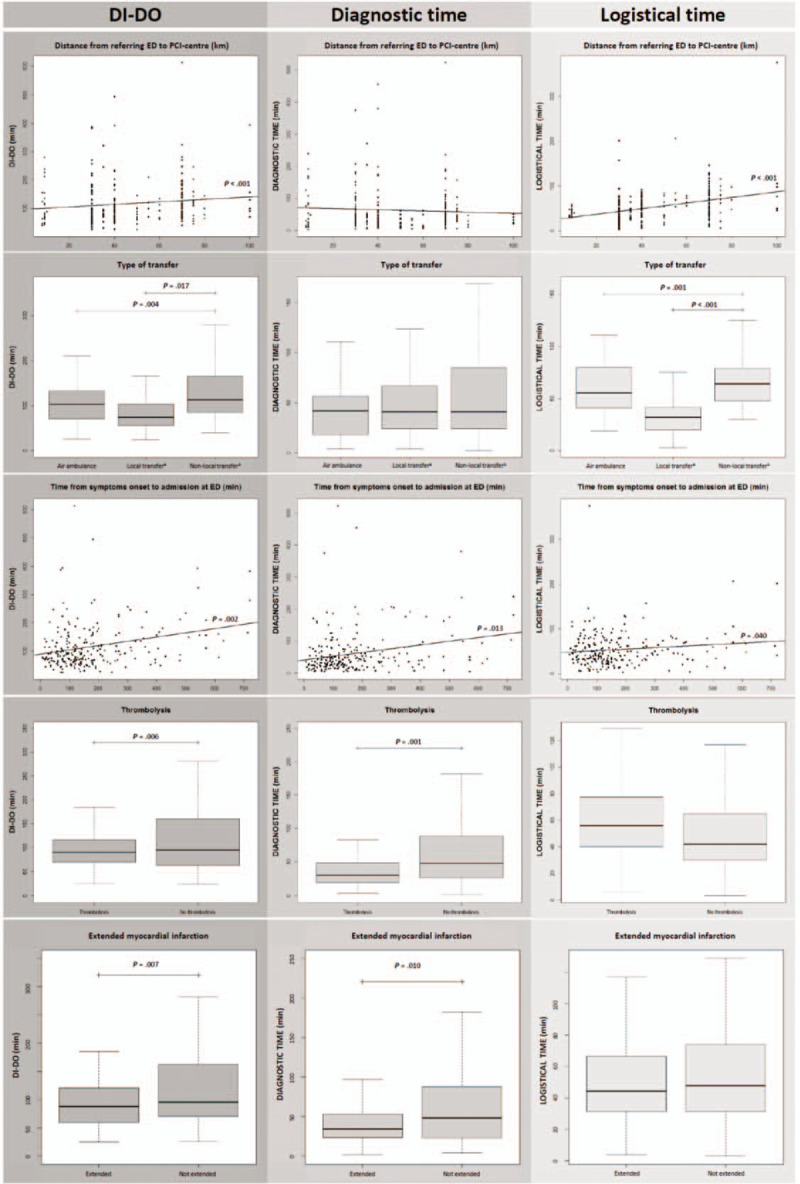
Scatter and box plots of the five main factors affecting door-in to door-out times. Only significant *P*-values are shown. Boxes show interquartile ranges; bold lines, medians; and whiskers, ranges. DI-DO = door-in to door-out, ED = emergency department, PCI = percutaneous coronary intervention. ^a^ Mobile intensive care unit (MICU) team available at the referring center; ^b^ MICU team had to come from another center.

Five major factors were associated with a shorter DI–DO time (Fig. 1): shorter distance between the referring ED and the PCI center (*P* < .001); transfer by air ambulance (*P* = .004) or local transfer (*P* = .017); shorter time from symptom onset to ED admission (*P* = .002); use of thrombolysis (*P* = .006); and extended myocardial infarction (*P* = .007). Supplemental Digital Content 6, contains details of all factors, whether associated or not.

Local transfer or transfer by air ambulance reduced the DI–DO time by improving logistical time (*P* < .001 and *P* = .001, respectively) (Fig. 1). A shorter distance between the referring ED and the PCI center reduced the DI–DO time by decreasing logistical time (*P* < .001). A shorter time from symptom onset to ED admission decreased the DI–DO time by decreasing diagnostic time (*P* = .013), with a linear link. The use of thrombolysis decreased DI–DO time by reducing the diagnostic time (*P* = .001). A diagnosis of extended myocardial infarction decreased the DI–DO time by reducing diagnostic time (*P* = .01).

## 4 Discussion

DI–DO times were longer than recommended in this retrospective observational French study of patients with acute STEMI who did not receive prehospital medical care and went direct to an ED without PCI facilities. Patients admitted direct to an ED without PCI facilities had a longer delay to reperfusion than those treated by the EMS in the same area.

Only 5/240 (2.1%) of our non-PCI center patients had a DI–DO time within the recommended ≤30 min.^[1]^ This is lower than in various studies from the United States and Canada, which have reported that 10% to 20% of patients achieved this target.^[12–15]^ Our median DI–DO time (92.5 min) also exceeded those in published studies from the United States and Canada, which ranged from 51 to 74 min.^[12–17]^ In two other US studies, median DI–DO times were reduced—from 83 to 68 min^[18]^ and from 97 to 58 min^[19]^ by making various changes to their protocols.

Among our non-PCI center patients, 57.9% underwent primary angioplasty, 32.4% within 120 min from first medical contact (admission to the ED) and the start of PCI (puncture of radial or femoral artery). This is considerably less than in a 2009 Canadian study, where 66.6% of STEMI patients benefited from primary angioplasty, 92% within the recommended 120 min.^[12]^

Although our rate of thrombolysis was high, it may not have been enough in our mountainous region. Only 43.0% of thrombolysed STEMI patients had signs of reperfusion on arrival at the PCI center. Therefore, plans to transfer patients to a PCI hospital for potential rescue angioplasty should start as soon as thrombolysis is initiated, as an urgent transfer.

The type of transfer was one of the most influential factors on DI–DO time. Patients who underwent local transfer had significantly shorter logistical times than those who underwent non-local transfer (*P* < .001). Similarly, US studies have reported that rural location significantly increased DI–DO times^[14]^ and that awaiting transport at the referring hospital was a common reason for delay.^[17]^ Transfer of the patient using an ambulance and MICU team provided by the local center is a critical point of care management to decrease transfer delays. However, less than half of the transfers were local. A shorter time from symptom onset to ED admission was also associated with reduced DI–DO (*P* = .002). Similar results have been reported in two studies from Canada.^[12,13]^ Other studies have reported that various factors are linked with shorter DI–DO times, including emergency transport to the ED,^[12,13,15]^ younger age,^[13,15,17]^ and male sex.^[12,14,15]^

The hospitals in our region should be organized to ensure the availability of urgent transfer of STEMI patients by a dedicated ambulance and MICU team in each ED in a center without PCI capabilities. Transfer by air ambulance should reduce transportation times in mountainous regions, such as ours. Although transfer by air ambulance did not significantly reduce DI–DO times compared to local transfer in our study, it likely decreases the delay to reperfusion as the transfer is faster. Overall, the choice of medical transportation from the referring ED to the PCI center should be the fastest available or with the shortest estimated first medical contact to balloon time.

### 4.1 Limitations

As a retrospective study, our data may have been affected by unknown factors associated with the DI–DO time (e.g., ED overcrowding, concomitant activity in the emergency call center, timing of the electrocardiogram, and delay to the first doctor's examination). Also, we did not analyse clinical severity or instability, which can affect the time spent in the ED. The sample size also precluded us from calculating odds ratios for patients with DI–DO ≤30 min. The findings may not be entirely representative of patients with acute STEMI, due to the logistical difficulties related to the geographic location. These findings may not be generalizable to other countries due to the specificities of the French EMS system.

## 5 Conclusions

In our regional French registry, 12.0% of patients with acute STEMI did not follow the dedicated prehospital pathway, and were thus admitted direct to an ED without PCI facilities. DI–DO times were long, with both diagnostic and logistical times exceeding 30 min, and only 2.1% of patients had a DI–DO time within the recommended ≤30 min. As nearly three quarters of patients with STEMI benefited from primary angioplasty beyond the recommended 120 min, we should aim for shorter DI–DO times and faster transfers, using an urgent local transfer (or the nearest MICU if there is no dedicated team at the site), or promote thrombolysis for eligible patients. However, current recommendations of DI–DO ≤30 min as a goal may not be realistic in everyday clinical practice.

## Acknowledgments

The authors would like to thank the RENAU network for the practical implementation of this registry and statistical analysis.

## Author contributions

**Conceptualization:** Sandrine Clot, Thomas Rocher, Vincent Descotes-Genon, Loic Belle.

**Data curation:** Sandrine Clot, Thomas Rocher, Mathieu Cardine, Mohamed Lotfi, Julien Turk, Pascal Usseglio, Vincent Descotes-Genon, Gerald Vanzetto, Dominique Savary, Guillaume Debaty, Loic Belle.

**Formal analysis:** Claire Morvan.

**Funding acquisition:** Sandrine Clot, Loic Belle.

**Methodology:** Sandrine Clot, Thomas Rocher, Loic Belle, Claire Morvan.

**Project administration:** Loic Belle.

**Resources:** Claire Morvan, Sandrine Clot, Thomas Rocher.

**Software:** Claire Morvan.

**Supervision:** Loic Belle.

**Writing – original draft:** Sandrine Clot, Thomas Rocher.

**Writing – review & editing:** Sandrine Clot, Thomas Rocher, Mathieu Cardine, Mohamed Lotfi, Julien Turk, Pascal Usseglio, Vincent Descotes-Genon, Gerald Vanzetto, Dominique Savary, Guillaume Debaty, Loic Belle.

## Supplementary Material

Supplemental Digital Content

## Supplementary Material

Supplemental Digital Content

## Supplementary Material

Supplemental Digital Content

## Supplementary Material

Supplemental Digital Content

## Supplementary Material

Supplemental Digital Content

## Supplementary Material

Supplemental Digital Content
