# Supplementary material for: Door-in to door-out times in acute ST-segment elevation myocardial infarction in emergency departments of non-interventional hospitals: A cohort study
Source: Medicine (Baltimore). 2020 Jun 5;99(23):e20434. doi: 10.1097/MD.0000000000020434 (PMC7306318; doi:10.1097/MD.0000000000020434)
Supplement: Supplemental Digital Content [file medi-99-e20434-s001.docx]

**Supplemental Digital Content 1**

The regional RESeau des URgences CORonaire (RESURCOR) registry area encompasses three main French administrative departments (Isere, Savoie, and Haute-Savoie) in the French Northern Alps. It covers 18 000 km², with an estimated population of 2 500 000, but with wide seasonal variation as a result of tourism. There are 19 emergency departments (EDs) without percutaneous coronary intervention (PCI) facilities () and three towns (Grenoble, Chambery, and Annecy) with a total of five PCI centres ( ). RESURCOR is part of a network that recommends a short delay to reperfusion; hence fast medical transfer (with a mobile intensive care unit [MICU] team) to a PCI centre is essential.^1^

**
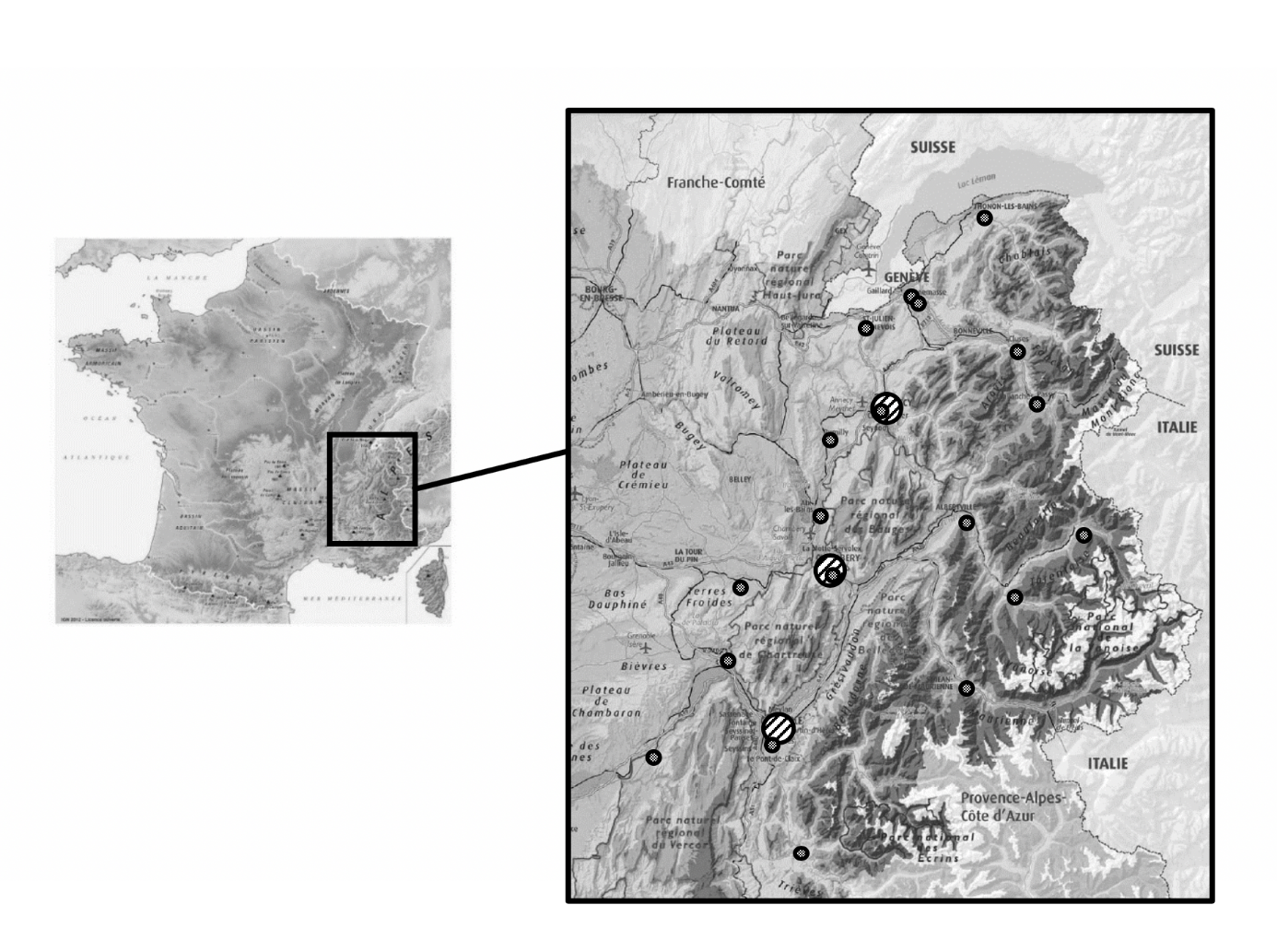
**

**1.** Belle L, Ageron F-X, Vallot C, RENAU. RECOMMANDATIONS RÉGIONALES POUR LA PRISE EN CHARGE DES SCA ST+ < 12 h. 2018; <https://www.renau.org/media/2018/03/1495-2018-stemi.pdf>. Accessed 20 Aug 2019.
