# Supplementary material for: Door-in to door-out times in acute ST-segment elevation myocardial infarction in emergency departments of non-interventional hospitals: A cohort study
Source: Medicine (Baltimore). 2020 Jun 5;99(23):e20434. doi: 10.1097/MD.0000000000020434 (PMC7306318; doi:10.1097/MD.0000000000020434)
Supplement: Supplemental Digital Content [file medi-99-e20434-s002.docx]

| **Supplemental Digital Content 2 Description of the emergency departments included** | | | | | | | | | |
| --- | --- | --- | --- | --- | --- | --- | --- | --- | --- |
| Centre | Annual admissions to ED (*n*) | Patients included (*n*) | MICU team available | Local ambulance available | Distance from PCI centre (km) | Time from PCI centre (min) | Transfer by air ambulance (*n*) | Local transfer^a^ (*n*) | Non-local transfer^b^ (*n*) |
| Hospital of Aix-les-Bains | 15 567 | 10 | Yes | Yes | 17 | 20 | 0 | 9 | 1 |
| Hospital of Annemasse | 20 921 | 12 | Yes | Yes | 37 | 27 | 2 | 6 | 4 |
| Hospital of Albertville | 52 779 | 36 | Yes | No | 56 | 35 | 0 | 27 | 9 |
| Hospital of Bourg-Saint-Maurice | 13 762 | 6 | Yes | No | 108 | 70 | 3 | 0 | 3 |
| Hospital of La Mûre | 7060 | 10 | No | No | 49 | 46 | 7 | 0 | 3 |
| Hospital of Moûtiers | 11 544 | 4 | Yes | No | 82 | 54 | 2 | 1 | 1 |
| Hospital of Le Pont-de-Beauvoisin | 15 810 | 4 | No | No | 36 | 35 | 0 | 0 | 4 |
| Hospital of Rumilly | 5372 | 1 | No | No | 27 | 28 | 0 | 0 | 1 |
| Hôpitaux du Pays du Mont-Blanc | 33 096 | 21 | Yes | No | 72 | 50 | 7 | 12 | 2 |
| Hospital of Saint-Jean-de-Maurienne | 14 059 | 13 | Yes | No | 79 | 51 | 5 | 2 | 6 |
| CHANGE Saint-Julien | 23 488 | 16 | Yes | No | 30 | 29 | 0 | 11 | 5 |
| Hospital of Saint-Marcellin | 7965 | 4 | No | No | 53 | 42 | 2 | 0 | 2 |
| Hospital of Thonon | 39 424 | 33 | Yes | No | 68 | 58 | 5 | 17 | 11 |
| Hospital of Voiron | 30 955 | 38 | Yes | Yes | 28 | 34 | 1 | 22 | 15 |
| Clinic les Cèdres | 22 244 | 17 | No | No | 12 | 13 | 0 | 0 | 17 |
| Clinic l'Espérance | 10 050 | 5 | No | No | 58 | 42 | 0 | 0 | 5 |
| Clinic Générale d'Annecy | 10 025 | 1 | No | No | 7 | 10 | 0 | 0 | 1 |
| Clinic Médipôle Savoie | 24 892 | 5 | No | No | 10 | 13 | 0 | 0 | 5 |
| Clinic HPPS | 22 905 | 4 | No^c^ | No | 39 | 29 | 0 | 2^c^ | 2 |
| TOTAL |  | 240 |  |  |  |  | 34 | 109 | 97 |
| ED, emergency department; MICU, medical intensive care unit; PCI, percutaneous coronary intervention.  ^a^ MICU team available at the referring centre.  ^b^ MICU team had to come from another centre.  ^c^ Although there was no MICU at this referring centre, there was a team at another hospital <5 minutes away, hence two transfers were considered local. | | | | | | | | | |
