# Supplementary material for: Door-in to door-out times in acute ST-segment elevation myocardial infarction in emergency departments of non-interventional hospitals: A cohort study
Source: Medicine (Baltimore). 2020 Jun 5;99(23):e20434. doi: 10.1097/MD.0000000000020434 (PMC7306318; doi:10.1097/MD.0000000000020434)
Supplement: Supplemental Digital Content [file medi-99-e20434-s003.docx]

**Supplemental Digital Content 3**

**Additional statistical methods**

Continuous variables included in the complete linear model were distance from referring emergency department (ED) to percutaneous coronary intervention (PCI) centre, duration of symptoms, age, systolic blood pressure, and heart rate. Categorical variables included sex, type of myocardial infarction (extended or anterior), cardiogenic shock, extreme consultation times (i.e. during weekends and night shifts), type of transfer, centre size, mobile intensive care unit (MICU) team and ambulance availability, thrombolysis performed, and year of myocardial infarction. To avoid model overload, stepwise regression and backward elimination of non-significant variables were used. Post-hoc analyses using Tukey’s honestly significant difference test were performed to determine statistical significance, when needed. The selected model for door-in to door-out (DI−DO) time was used for both diagnostic time and logistical time.
