# Supplementary material for: Door-in to door-out times in acute ST-segment elevation myocardial infarction in emergency departments of non-interventional hospitals: A cohort study
Source: Medicine (Baltimore). 2020 Jun 5;99(23):e20434. doi: 10.1097/MD.0000000000020434 (PMC7306318; doi:10.1097/MD.0000000000020434)
Supplement: Supplemental Digital Content [file medi-99-e20434-s004.docx]

**Supplemental Digital Content 4**

Flow chart of the study population.; RESURCOR: RESeau des URgences CORonaire (a regional registry in the French Northern Alps that analysed data from patients with ST-segment elevation myocardial infarction [STEMI] of duration <12 hours); MICU: mobile intensive care unit; ED: emergency department.

RESURCOR 2012–2014

*N*=2007

Prehospital medical care by MICU

*n*=1416

Hospitalized for another medical condition and who developed STEMI, *n*=56

Admitted directly to intensive care unit

*n*=12

Early cardiac arrest (before ED)

*n*=98

ED without catheterization facilities

*n*=240

ED with catheterization facilities

*n*=185

Admitted to ED without medical care

*n*=425
