# Supplementary material for: Door-in to door-out times in acute ST-segment elevation myocardial infarction in emergency departments of non-interventional hospitals: A cohort study
Source: Medicine (Baltimore). 2020 Jun 5;99(23):e20434. doi: 10.1097/MD.0000000000020434 (PMC7306318; doi:10.1097/MD.0000000000020434)
Supplement: Supplemental Digital Content [file medi-99-e20434-s005.docx]

| **Supplemental Digital Content 5 Baseline and treatment characteristics according to initial care** | | | |
| --- | --- | --- | --- |
|  | Prehospital medical care by MICU team (*N*=1416) | Admitted directly to ED (*N*=425) | *P*-value |
| Age (years) [median (IQR)] | 63 (53–75) | 61 (51–72) | .012 |
| Men [*n* (%)] | 1098 (77.5) | 315 (74.1) | .16 |
| Presenting characteristics [median (IQR)] |  |  |  |
| Systolic blood pressure (mmHg) | 140 (122–160) (*n*=1374) | 145 (125–162) (*n*=407) | .039 |
| Heart rate (beats/min) | 75 (64–88) (*n*=1357) | 76 (66–90) (*n*=399) | .012 |
| Cardiac history [*n* (%)] |  |  |  |
| History of myocardial infarction | 237 (16.7) | 40 (9.4) | < .001 |
| Previous PCI | 188 (13.3) | 32 (7.5) | .002 |
| Previous CABG | 25 (1.8) | 5 (1.2) | .53 |
| Diabetes [*n* (%)] | 203 (14.3) | 56 (13.2) | .60 |
| Cardiogenic shock [*n* (%)] | 52 (3.7) | 10 (2.4) | .24 |
| Anterior myocardial infarction [*n* (%)] | 581 (41.0) | 180 (42.4) | .67 |
| Extended myocardial infarction [*n* (%)] | 518 (36.6) | 123 (28.9) | .005 |
| Left bundle branch block [*n* (%)] | 28 (2.0) | 3 (0.7) | .12 |
| Limited ST-segment elevation^a^ [*n* (%)] | 35 (2.5) | 11 (2.6) | 1.00 |
| Cardiac arrest after first medical contact [*n* (%)] | 84 (5.9) | 8 (1.9) | .001 |
| Reperfusion [*n* (%)] |  |  |  |
| Thrombolysis | 278 (19.6) | 81 (19.1) | .85 |
| Primary angioplasty | 999 (70.6) | 311 (73.2) | .22 |
| None | 139 (9.8) | 33 (7.8) | .24 |
| Management delay (min) [median (IQR)] |  |  |  |
| Time from symptom onset to first medical contact | 87 (52–169) (*n*=1361) | 126 (77–217) (*n*=401) | < .001 |
| Time from symptom onset to thrombolysis | 100 (72.75–150) (*n*=275) | 137 (90–200) (*n*=81) | < .001 |
| Time from admission to thrombolysis | 22 (15–30) (*n*=275) | 36 (25–47) (*n*=81) | < .001 |
| Time from initial ED admission to primary angioplasty^b^ | 70 (56–92) (*n*=968) | 104 (61–169) (*n*=310) | < .001 |
| MICU, mobile intensive care unit; ED, emergency department; IQR, interquartile range (25th to 75th percentiles); PCI, percutaneous coronary intervention; CABG, coronary artery bypass graft.  ^a^ <1 mm.  ^b^ First medical contact to balloon. | | | |
