# Supplementary material for: Door-in to door-out times in acute ST-segment elevation myocardial infarction in emergency departments of non-interventional hospitals: A cohort study
Source: Medicine (Baltimore). 2020 Jun 5;99(23):e20434. doi: 10.1097/MD.0000000000020434 (PMC7306318; doi:10.1097/MD.0000000000020434)
Supplement: Supplemental Digital Content [file medi-99-e20434-s006.docx]

| **Supplemental Digital Content 6 Factors associated with reduced door-in to door-out time** | | | |
| --- | --- | --- | --- |
| Variable | *P*-values | | |
|  | DI–DO time | Diagnostic time | Logistical time |
| Distance from referring ED to PCI centre | < .001 | .072 | < .001 |
| Type of transfer (air ambulance, local^a^, or non-local^b^) | < .001 | .10 | < .001 |
| Time from symptoms onset to admission to ED | .002 | .013 | .040 |
| Use of thrombolysis | .006 | .001 | .68 |
| Extended myocardial infarction | .007 | .010 | .39 |
| Anterior myocardial infarction | .048 | .015 | .64 |
| Size of centre (assessed by number of annual admissions to ED) | .14 | .06 | .64 |
| Heart rate | .07 | .11 | .37 |
| Year | .33 | .51 | .56 |
| Available ambulance | .99 | .47 | .15 |
| DI–DO, door-in to door-out; ED, emergency department, PCI, percutaneous coronary intervention.  ^a^ MICU team available at the referring centre.  ^b^ MICU team had to come from another centre. | | | |
